# An extensible framework and database of infectious disease for biosurveillance

**DOI:** 10.1186/s12879-017-2650-z

**Published:** 2017-08-07

**Authors:** Ashlynn R. Daughton, Reid Priedhorsky, Geoffrey Fairchild, Nicholas Generous, Andrea Hengartner, Esteban Abeyta, Nileena Velappan, Antonietta Lillo, Karen Stark, Alina Deshpande

**Affiliations:** 1 Los Alamos National Laboratory, Los Alamos, USA; 2 Digital Infuzion, Inc., Gaithersburg, USA

**Keywords:** Biosurveillance, Infectious disease, Disease hierarchy, Disease surveillance, Disease ontology, Disease database

## Abstract

Biosurveillance, a relatively young field, has recently increased in importance because of increasing emphasis on global health. Databases and tools describing particular subsets of disease are becoming increasingly common in the field. Here, we present an infectious disease database that includes diseases of biosurveillance relevance and an extensible framework for the easy expansion of the database.

## Background

Biosurveillance is a relatively young field. While the first health surveillance systems are from the fourteenth and fifteenth centuries during the Black Death (a large outbreak of plague) [1], health surveillance was only recognized as its own field in the 1960s [1], and the United States’ first national strategy for biosurveillance was released only in 2012 [2]. Further, this discipline is broad in nature. The national strategy for biosurveillance calls for systems to “detect, track, investigate, and navigate incidents affecting human, animal, and plant health, thereby better protecting the safety, well-being, and security of the American people” [2].

Because of the breadth that human, plant and animal health encompasses, only recently has there begun to be consensus in the field about what the full “biosurveillance” spectrum is, what data streams are included in such surveillance, and further, what diseases are relevant. An extensive review of the definition and breadth of biosurveillance is available in Margevicius et al. [3]. This work was used to develop the Biosurveillance Resource Directory (BRD), a database of resources with biosurveillance relevance including disease surveillance reports, epidemiological models [4], and related organization and contact information [3]^1^. Because the scope of biosurveillance is broad, the BRD includes resources for infectious diseases affecting human, plant, and animal populations, as well as sentinel surveillance systems that capture syndromic definitions of infectious disease. Surveillance systems range from laboratory based systems where samples are collected and processed (e.g., FluNet [5]), to systems that scrape news media and look for evidence of disease outbreaks (e.g., HealthMap [6]). The diseases included in the purview of each system differ substantially. For example, because ProMED is scraping news data worldwide, they are able to collect information on a vast number of illnesses. Other systems have more focused agendas; FluNet, a system provided by the World Health Organization (WHO), focuses exclusively on influenza.

In order to fully describe each system in the BRD, an unambiguous description of the relevant infectious diseases and/or syndromic categories of relevance was required. There are a handful of databases and ontologies currently available that pertain to disease: the Diseases Database [7], the Disease Ontology [8–10] and the Infectious Disease Ontology [11, 12]. These were initially surveyed as possible ways to describe diseases in the BRD. While the databases provide rich schemas, they did not provide the relevant descriptions we required (for reasons described below).

The Diseases Database is described as an “inhouse search engine” [13] and includes diseases, drug names, and symptoms. It is a self-described “limited and idiosyncratic subset” [7], but does contain several thousand terms, including many disease synonyms. However, there is no method to download or export the data and they request that others refrain from scraping information.

The Disease Ontology and the Infectious Disease Ontology are formal ontologies of human disease. The Disease Ontology captures human disease broadly, including infectious diseases, various noncommunicable diseases (e.g., cardiovascular diseases), and genetic diseases [9]. It additionally connects various disease vocabularies [9]. However, as described by Cowell and Smith [12], there are some issues with the implemented hierarchy classification that result in inconsistent groupings of diseases. The Infectious Disease Ontology provides information for the more narrow field of infectious disease [11, 12]. There are a number of extensions of this ontology for specific diseases, and diseases with specific transmission groups. However, while there is a disease hierarchy, there is no inclusion of syndromic categories, and the number of diseases with extensive ontologies are limited. Further, both the Infectious Disease Ontology and the Disease Ontology are focused on human disease, and are developed largely with genetic biomedical data in mind. While genetic and biomedical data are important, they have less relevance in population level health, because genetics and specific disease symptoms tend to vary among individuals. As biosurveillance tends to be concerned with outbreaks at a population level, descriptions of the disease at a high level (e.g., transmission routes, hosts, causative agents etc.) are more useful than, for example, descriptions of which particular tissues are infected by the disease.

Because of these differences in scope, our team decided to develop a new database that systematically describes infectious diseases from a population-based public health focus. Further, because the BRD includes resources that track disease in multiple populations (human, plant and animal), the framework was designed with extensibility in mind. The remainder of this paper will discuss the resulting classification system developed to describe these diseases.

## Construction and content

As discussed briefly above, descriptions of disease with respect to biosurveillance differ in important and systematic ways from the previous biomedically related frameworks. Our team identified a set of seven requirements for the database. They are:

- **Correctly identify diseases from synonyms**: *German measles*, for example, is not a term for measles, but rather for the disease rubella. Similarly, *rubeola* refers not to rubella, but to measles [14]. It was vital to ensure that our database capture these synonyms, and others like them, without confusion. Further, much of the current work organizing diseases occurs in English. However, those in biosurveillance speak a variety of languages. Thus, the capacity to include synonyms in other languages is also important.
- **Describe transmission** of the disease. High level information about the way the disease is transmitted is necessary. Many diseases are capable of multiple modes of transmission. For example, anthrax can be air-borne, acquired by contact with an infected animal, or, in rare cases, ingested and transmitted through contaminated meat products [15]. The database should inclusively describe all routes of transmission. If one mode of transmission is via a vector, that organism should be clearly described as well (see next bullet).
- **Describe related organisms (e.g., causative agent, hosts and applicable vectors)** of the disease. Organisms are associated with a disease in three ways: causing, spreading, or being infected with the disease. Organisms should be described at varying levels of resolution, based on available data. For example, snthracnose is a disease that affects plants broadly [16], whereas apple Scab specifically affects apple tree [17]. A search for “plant” diseases (i.e., diseases where plants are the host) should return both diseases. However a search for “apple” diseases, should only return the latter. Similar principles apply for causative agents and vectors. Some diseases, such as dengue and chikungunya, are spread by specific vectors, in this case, *Aedes aegypti* and *Aedes albopictus* [18]. Other diseases, for example, avian pox, are transmitted by “mosquitoes” more generally [19]. A user searching for all “mosquito” diseases should find those with the generic term “mosquito” as the vector, as well as any that list specific species of mosquitoes.
- **Flag items of biosurveillance relevance** to particular sub-fields. Within biosurveillance, resources focus on particular subsets of disease. Some, for example, focus on bioterrorism (e.g., BioALIRT [20]^2^), while others focus on reportable diseases (e.g., 122 Cities Mortality Reporting System [21]). In order to maximize utility, we wanted to be able to aggregate diseases that fell under particular categories, as well as diseases that fell within multiple categories. Current categories are: bioterrorism diseases, diseases of economic importance, the United States’ reportable diseases, vaccine-preventable diseases, zoonotic diseases, drug-resistant diseases, and emerging or re-emerging diseases. This list may not be exhaustive. It is also important to be able to broaden the scope in the future if needed.
- **Specify disease information in varying levels of detail**: Much of biosurveillance occurs as syndromic surveillance [22]. Such systems look for particular clinical symptoms, or syndromes, rather than for confirmed diagnosis of particular diseases. Thus, it was also important that we be able to understand the links between syndromes and diseases.
- **Be extensible**: It became clear early on that any biosurveillance database would need to be easily extensible to other data, and potentially to other languages. Thus, the goal was to provide a framework that was simple and useful enough to extend in other directions as it became necessary. We also noted that, while our team works predominantly in English, many in the field of biosurveillance do not. Because disease names and synonyms change with language, it was important that the resulting framework be extensible to other languages.
- **Be transparent**: Because information about some diseases may be contested, it is imperative that all source documentation be explicit such that users could verify data provenance easily.

In addition to the above domain requirements, we wanted to develop a technical framework that could be easily applied to biosurveillance tools and web-applications. We thus specified two specific technical requirements:

- **Variety of formats available**: Describing information in a human and computer-readable form can be complicated. Numerous frameworks exist to do this. The benefits and complexities of each are outside the scope of this paper, but we will describe a few with particular relevance. Resource Description Framework (RDF) is one such framework that is used to describe things in a computer-readable format. It is commonly used in conjunction with eXtensible Markup Language (XML), a markup language that has associated rules to govern its structure. These rules describe how data can be represented. The combination of these two (RDF/XML) is commonly used to describe ontologies (OWL format). The combination provides a mechanism for describing semantic information (like hierarchies and relationships between concepts). However, they are predominantly used by ontologists. Other formats (e.g., only XML or JavaScript Object Notation (JSON)), are more commonly used to transfer information between web-based applications. Rather than restrict this database to an OWL format (as the ontologies cited have chosen to do), we wanted to design our database to allow more exportation in a variety of formats to enable easy use with different tools and applications. Further, for users that would like to directly interact with the data, we also stressed the importance of a user interface.
- **Application Program Interface (API)**: It was also important to have an easy mechanism to query and use the database. One such mechanism is an Application Program Interface (API). API’s allow other programs to retrieve database results in one of the computer-readable formats described above. Including an API allows for easy interactions between databases, or to other online tools.

### Database construction

The database is built using PostgreSQL [23], a relational database management system, and Django [24], a framework to develop web-based applications. In this database, information is contained in tables that can have relationships and allow the characterization of disease along many axes. Currently, we use the following terms to describe each disease:

- Agent: This is the causative agent of the disease. For example, *Plasmodium vivax* is a causative agent of malaria.
- Population: This is the population the disease affects. For example, malaria affects humans. Carrier hosts (symptomatic and asymptomatic) are also included in this population.
- Disease synonym: These are names referring to the same disease. For example, malaria is sometimes referred to as *Malignant tertian fever*.
- Property: These are flags of biosurveillance relevance. Malaria is flagged as drug resistant, emerging or re-emerging and a US notifiable disease.
- Transmission: This is the mechanism for transmission of the disease from one population member to another. Options are binned into air-borne, casual contact, fomite, ingestion, in-utero, sexual transmission, vector-borne and water-borne.
  - Vector-borne diseases include another field for the vector. This is an organism that helps transmit the disease. It is only present in vector-borne diseases. In the case of malaria, the vector is the *Anopheles* mosquito.
- Disease parent: This is used to show hierarchical relationships between diseases or disease categories (described in more depth below). For example, malaria, has the syndromic group *febrile illness* as the parent.

**Fig. 1:**
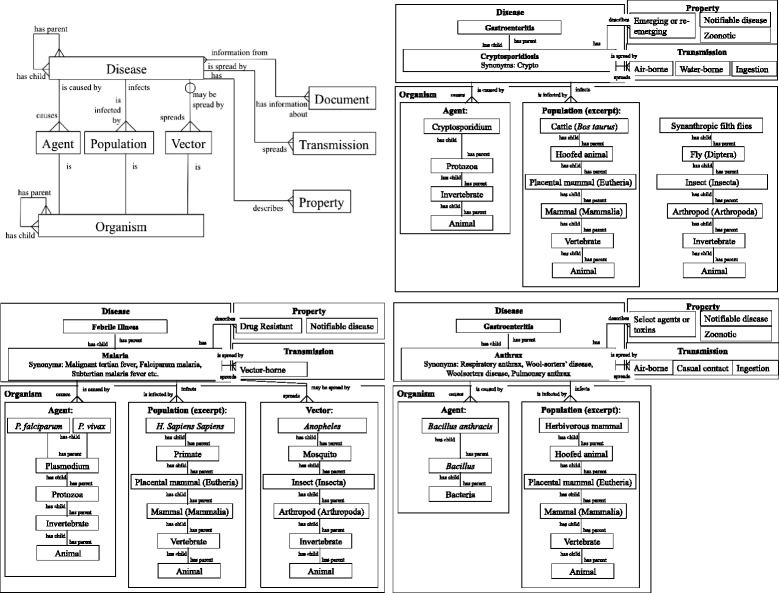
Database structure and corresponding example. Entity relationship diagram for the database. Disease has 6 main descriptors: agent, population, vector, property, transmission and document. Organisms (agents, populations and vectors) are described by common and scientific names and include a hierarchical component. Transmission and property are categorical lists with relevant terms and associated descriptions. Document describes source information. Diseases are described by their 6 components as well as through their disease hierarchy. Connecting symbols describe the type of relationship: three prongs describe many-to-many relationships, *straight lines* indicate a one-to-one mapping, and the *line with open circle* describes a relationship than can be present but does not have to be. This structure with respect to malaria is shown in the second half. Documents have been omitted and some organism associations were truncated for brevity. Both organisms and diseases have hierarchy elements, allowing for optimal searching and more complete disease descriptions. Diseases are described by associated synonyms, properties and transmission

A visual representation of the current schema of our database is in Fig. 1. The relationships between disease and disease attribute tables (e.g., organism, property, transmission) are described, as well as relationships to the document tables that are used throughout the BRD to track data provenance. Relationships between tables are described by the symbol and words used to link the tables (see figure caption for more information).

There are multiple ways organisms are important to a disease’s description including the population affected, the agents which cause the disease, and, if applicable, the vectors that spread the disease. Further, the framework allows tables to be self-referencing, or to have hierarchies. For example, some diseases in the database affect “mammals” generally, while others affect a specific mammal (e.g., *Homo sapiens*). In the latter example, the database also allows an organism parent, such that *Homo sapiens* is listed as a child of mammals. Any particular organism can then be related to a particular disease attribute. This allows a user to query fields at multiple levels of specificity. A user can identify all diseases that affect “mammals” or all disease than affect humans, specifically. This is true for all organism fields: agent, population and vector.

Just as organisms have self-referencing ties allowing a hierarchy, so do diseases. Our disease hierarchy has two components. The first is that some clinical diseases are parents of other diseases. For example, influenza is a parent of avian influenza A. The second is that diseases also fall into syndromic categories that are treated like diseases, but are flagged as syndromes. Influenza, in this case, is also a child of “respiratory diseases”. The parent-to-child relationship is a many-to-many one, meaning that diseases can be the children of multiple parents, and vice versa. This allows for broad specification of disease.

There are a variety of schemas to describe syndromic categories of disease, however they tend to have large overlap. For the purposes of this database we used a modification of the Centers for Disease Control and Prevention’s (CDC) Essence II categories [25]. Specifically, we use: respiratory, gastrointestinal, febrile, hemorrhagic, dermatologic, and nervous system.

From previous work describing the breadth of biosurveillance [3], we identified common categories of specific interest in the field and incorporated these as flags for relevant diseases. Flags currently include select agents and toxins, diseases of economic importance, reportable diseases (United States), vaccine-preventable diseases, zoonotic diseases, drug resistant diseases, and emerging or re-emerging diseases, but can be expanded as necessary.

A specific example of the database structure with respect to malaria, anthrax and cryptosporidiosis is given in Fig. 1. Relationships between organism, agent, population, vector (if applicable), and their respective associations to the disease are described, as well as relationships between disease and disease syndrome, and disease and properties/ transmission.

### Database content

The diseases currently included in our database were manually curated, beginning with the United States’ list of notifiable diseases, and the infectious diseases included in the Disease Ontology. The list was then expanded based on the human, plant and animal diseases included in surveillance systems in the BRD. Possible synonyms for diseases were initially identified using WordNet [26, 27]. Associated disease metadata was collected through extensive literature review, during which time additional synonyms were also added. The first author curated the initial information, The other authors with expertise in biology and infectious diseases verified accuracy. Each disease was reviewed by at least two co-authors. All citations used to identify data are included, so provenance is completely transparent. This protocol is extremely time consuming, and is probably not feasible for a larger collection. Intelligent automation of portions of this procedure are an active area of interest.

## Utility and discussion

### User and API interfaces

**Fig. 2:**
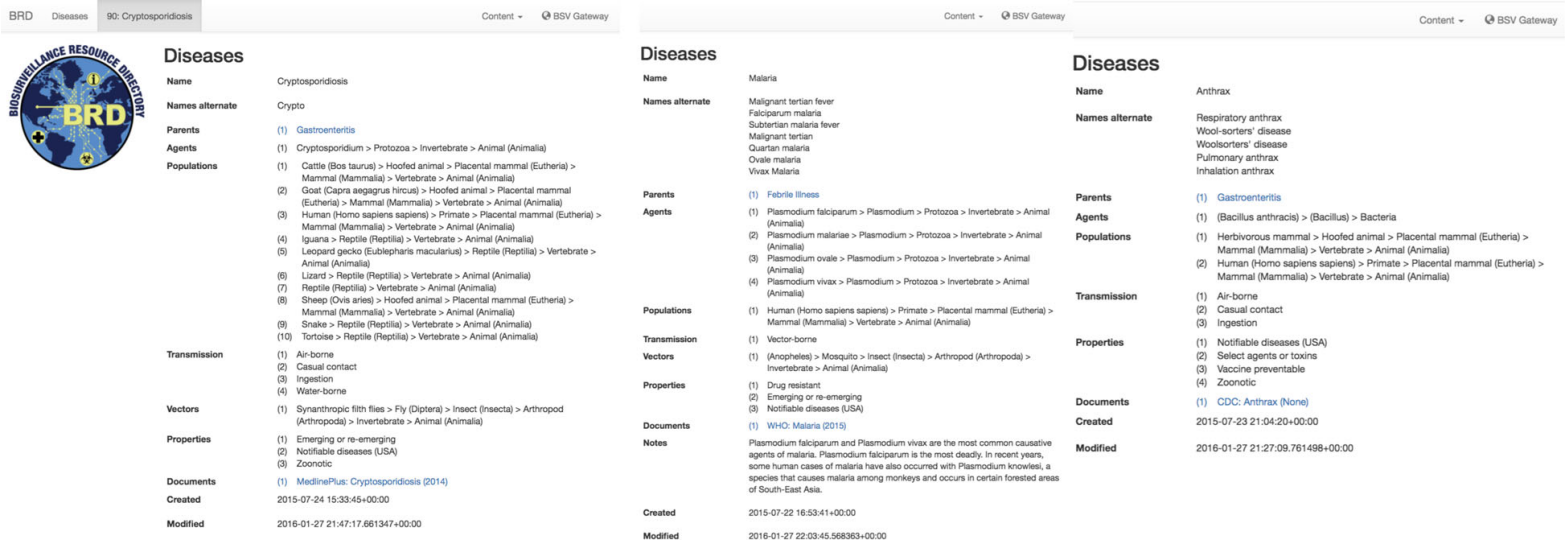
Example of malaria, anthrax and cryptosporidiosis as they appear in the database. Names, synonyms, parents, associated organisms (agents, vectors, and populations) and sources (documents) are shown. Letters in *blue* are links to other database elements containing more information (e.g., “Gastroenteritis” in anthrax)

Django allows the development of a simple front-end interface (see examples in Fig. 2). This interface allows a user to search the database, see connections between diseases and related surveillance systems, find information about the disease, and see where the information was obtained from. In addition to the front-end interface, we implemented a REST API using Django’s REST API framework [28]. This allows users to query the database and export to JSON and XML. Further, we designed an export of the database to RDF/XML compatible with OWL, the format currently utilized by ontologists. Our own biosurveillance tools^3^ take advantage of the database and the API. Others, may choose to take advantage of other formats (e.g., RDF/XML), as needed. Of note, references are not currently included in exports, or as part of the API.

### Utility for other applications

Using the above methods we have characterized 280 diseases encompassing 69 animal diseases, 70 human diseases, 55 plant diseases, and 63 diseases that affect both human and animal (i.e., zoonotic). Figure 2 shows the web-application interface for three such diseases as an example. Both the name and possible alternate names are shown, in addition to the hierarchical disease parent, and all relevant organisms. Organisms are classified from the most specific information collected (e.g., *Bacillus anthracis*) and shows all organism parents (e.g., *Bacillus*). Names are classified both as common names (e.g., human) or as scientific names using parentheses (*Homo sapiens sapiens*). This particular example illustrates a disease with varying levels of organism knowledge. For example, the causal agent is known to the species level, but an exhaustive list of possible populations that could be infected by anthrax was not available in literature. Thus we have specified humans, as well as “herbivorous mammals”.

Using this database, we have associated specific diseases, or types of diseases, with relevant biosurveillance resources and disease models in the Biosurveillance Resource Directory [3]^4^. The anthrax example has 29 associated biosurveillance resources including various ministries of health, and several animal health networks. This allows a user to precisely identify which diseases are related to particular biosurveillance systems and vice versa.

### Limitations

Describing diseases in a useful, extensible, but detailed manner is difficult. We recognize several specific limitations in the current design of our database.

First, it is important to note that there are numerous ways to classify disease relationships, and that the appropriate classification of relationships between diseases is difficult and can depend upon context and application. Different types of influenza, for example, can be classified based on their surface glycoproteins (typically includes Influenza A), or based on their lineage and strain (typically includes Influenza B) [22, 29]. Other viruses are classified based on morphology [30], the location where the first recognized outbreak occurred (e.g., ebola) [31], or other metrics entirely.

Within the field of biosurveillance, this difficulty manifests itself in specific ways. Most surveillance systems are broad enough that they do not discriminate based on subcategories of illnesses (i.e., a surveillance system is likely to include all ebola viruses, not restrict to particular strains). However, those same surveillance systems often want to track the subcategories of common illnesses to discover and study important epidemiological trends. Thus, a correct hierarchy is important in this database.

Currently, most of the diseases included have straightforward parent-child relationships. Most diseases are included in a syndromic category, but have few if any relationships with other diseases. Influenza is the current exception, where there are some subcategories, including “avian Influenza A” and “Swine Influenza”. The next iteration of the database should be expanded to include more specific relationships (e.g., influenza A H5N1 as a child of “avian influenza A”). We plan to follow standard practice for hierarchies, based off practices accepted in literature (e.g., influenza B will be described by lineages, and influenza A by glycoproteins). It is highly likely that situations will arise where a child might belong to multiple subcategories. Fortunately, the current database architecture makes relationships like that quite simple. Hierarchies can also be refined as epidemiological practices change.

Second, requirements for this database were identified through our team’s specific needs with respect to other biosurveillance tools. We believe this framework and the resulting database are useful, more broadly. However, it is possible that our list of requirements was not exhaustive. As additional work is done in this field requirements will likely be modified and added. The framework built supports such extension. Interview-based studies with surveillance system users, public health analysts, and epidemiologists would be of tremendous use in this capacity.

Third, diseases are currently not associated with particular geographic locations. Geospatial analyses are hugely important to disease surveillance, especially as diseases emerge, re-emerge, develop various types of antibiotic resistance etc. However, associating disease with specific locations can also be difficult, because it inherently requires some temporal association. For example, a geographic field could describe if (1) the disease had ever been present, (2) the disease had been present within the past *N* years, (3) the disease is currently present, or if (4) this disease was projected to be present soon (within *N* years). All of these might provide useful information, but designing the related database components requires careful thought.

Last, the current process for developing this database relies substantially on manual curation by a team of biologists and public health experts. That has allowed us to put a level of detail into the database that we believe is beneficial. However, we also recognize the substantial number of hours required to maintain the database.

## Conclusions

Future work will aim to address the limitations described above, to the extent possible. Additional work might include the expansion of the database to include new information. For example, the addition of epidemiological variables (e.g., the reproductive number, infectiousness period) may be of use to the disease modeling community. Other useful additions might be synonyms in additional languages, or International Classification of Disease (ICD) codes. Mapping relevant ICD codes to diseases would allows users to identify relevant codes to use for case definitions, a common practice for epidemiological studies (e.g., [32]).

There is also room for addition of more query capabilities within our API that would result in more comprehensive app-to-app communication. Additional next steps include the set up of a public repository for version tracking and to allow outside contributors to make suggestions for content. We believe a community effort for the maintenance of this tool will improve the content and breadth overall.

## Availability and requirements

**Project name:** Disease Database; Biosurveillance Resource Directory**Project home page:** https://brd.bsvgateway.org/ **Operating system:** OS-agnostic

## Endnotes

^1^ See brd.bsvgateway.org.

^2^ See http://brd.bsvgateway.org/system/35/.

^3^ For example see aido.bsvgateway.org.

^4^ Available at brd.bsvgateway.org.

## Abbreviations

API: Application Program Interface
BRD: Biosurveillance Resource Directory
CDC: Centers for Disease Control and Prevention
ICD: International Classification of Diseases
JSON: JavaScript Object Notation
RDF: Resource Description Framework
SME: Subject Matter Expert
WHO: World Health Organization
XML: eXtensible Markup Language
